# The influence of students’ sense of social connectedness on prosocial behavior in higher education institutions in Guangxi, China: A perspective of perceived teachers’ character teaching behavior and social support

**DOI:** 10.3389/fpsyg.2022.1029315

**Published:** 2022-11-23

**Authors:** Nanguang Su, Hsuan-Po Wang

**Affiliations:** ^1^Dhurakij Pundit University, Bangkok, Thailand; ^2^School of Architecture and Electrical Engineering, Hezhou University, Guangxi, China

**Keywords:** higher education students, perceived social support, teacher character teaching behavior, prosocial behavior, sense of social connectedness

## Abstract

The objective of this study is to examine the sense of social connectedness (SSC) and prosocial behavior (PB) of students in Guangxi higher education institutions in China and to further understand the factors influencing PB of higher education students. In this study, a total of 1,007 students were sampled from 8 Guangxi higher vocational schools through purposive sampling using questionnaires, of which 676 (67.1%) were male students and 331 (32.9%) were female students. This study further enriches self-determination theory by exploring the effects of teachers’ character teaching behaviors and social support on PB, using the SSC as an intrinsic motivation. In addition, the results of the study revealed that SSC, perceived teacher character teaching behaviors and perceived social support (PSS) were positively related to students’ PB. SSC affects PB not only directly, but also indirectly through the mediating role of perceived teacher character teaching behavior and perceived teacher support. Teacher character teaching behaviors and PSS also play a chain mediating role in the relationship between SSC and PB. Finally, this study provides strategies to optimize school character management for higher education students in order to meet their basic psychological needs and thus promote the production of PB.

## Introduction

Prosocial behavior (PB) plays a crucial role in increasing well-being, improving interpersonal communication and promoting harmonious social development (Rose et al., 2022; Su et al., 2019). Especially for students entering higher education, PB is crucial for students’ interpersonal relationships, psychological well-being, subjective well-being and social adjustment (Meehan et al., 2019; Son and Padilla-Walker, 2020; Nagaoka et al., 2022). PB has become an important hotspot in the field of organizational behavior and a series of research themes have been developed (Xiaoping et al., 2019). However, despite much academic work on PB, there is still a lack of attention to adolescent PB (Shin and Lee 2021). In particular, there is a large gap between the students trained in vocational schools and the needs of society, and there is growing concern about the less PB that vocational students exhibit after employment (Lv et al., 2022). In addition, undesirable phenomena such as campus bullying, depression, and poor learning atmosphere are common in Chinese higher education institutions (Xu et al., 2022). The current Chinese education system, which focuses more on students’ course performance and neglects character development and PB motivation (Liu and Zhang, 2021), which makes the progress of vocational education in China a great challenge. Thus, this study seeks to find the effect mechanism of PB by taking higher education students as the research participant to further fill the research gap of related topics.

Weaver et al. (2022) noted that people have a basic need to belong and that people who have an enhanced sense of connectedness with each other show less tendency to be aggressive. Therefore, by facilitating connectedness is also an effective way to achieve social benefits (Pavey et al., 2011; McLoughlin and Hermens, 2018). Armstrong-Carter et al. (2021) suggested that strong prosocial tendencies arise when adolescents feel highly motivated to connect with others. Van Ryzin et al. (2020) also verified that more positive peer relationships contribute to higher levels of PB through a study of the relationship between cooperative learning and PB. Furthermore, when students have a strong sense of connectedness to society, they produce more PB to improve their image (Chen and Jordan, 2018). It has also been suggested that social connectedness can buffer adolescents from the negative effects of discrimination or exclusion (Arslan, 2018), mitigate the risk of domestic and school violence (Valido et al., 2020), and avoid health risk behaviors such as excessive alcohol consumption (Montal-Rosenberg et al., 2022). Conversely, social exclusion leads to a marked increase in what is considered violent and aggressive behavior (Debono et al., 2020), as well as a decrease in cooperative and PB (Schaan et al., 2020; Quarmley et al., 2022). In conclusion, positive social connectedness and normative peer groups can increase the production of PB (Malonda et al., 2019).

Teachers are role models and role models for their students. In schools, teachers have the most contact with students and influence their development in different settings at all times. For example, classroom teaching, daily life actions and words (Gunawan, 2019). Furthermore, teachers’ character teaching affects students’ beliefs, values, attitudes toward school and students’ future achievement, i.e., good teacher character teaching behaviors play a very important role in students’ growth (Lian et al., 2020). Thus, it becomes especially critical to improve teachers’ character teaching behaviors. Pritchard et al. (2018) argued that teachers are not only educational providers, but can also build relationships with the characters they interact with. When students have a stronger sense of social connectedness (SSC) and are more connected to their teachers, teachers exhibit more character teaching behaviors such as appreciation and support (Maloney and Matthews, 2020). Conversely, students’ poorer SSC can lead to deterioration in teacher-student relationships, leaving teachers emotionally exhausted and frustrated and frequently using punitive and reactive behaviors (Oberle et al., 2020), and such negative teacher behaviors are detrimental to the production of PB in students (Van Ryzin et al., 2020). Therefore, this study concludes that higher education students’ SSC has a significant impact on teachers’ character teaching behaviors.

It has been shown that feelings of being supported by others play an important role in promoting positive psychological aspects of oneself (Newman et al., 2018). Furthermore, close social relationships lead to more social support (Davis and Kiang, 2016), and that the social support received in turn promotes a SSC, as connected communication between individuals can meet people’s social relationship and others to build emotional connectedness (Hashemi et al., 2020). Positively related to students (PSS) refers to one’s perception of whether the people around him or her are supportive (Xie et al., 2022). Many theoretical and empirical studies (Hashemi et al., 2020; Çiçek, 2021; Karabatak and Alanoğlu, 2021) have shown that social connectedness is related to PSS, such as a positive relationship between social support and coming from family, friends and social connectedness (Robinson et al., 2020). In this sense, the social connectedness that individuals make with others increase people’s confidence in receiving social support in times of need.

Teachers are important for student development, as they help students develop positive responses and promote PB (van den Bos et al., 2018). In schools, teachers are central to education, key determinants of student progress, and can have both positive and negative effects on students (Fogelgarn et al., 2021). In addition, teachers character teaching behaviors heavily influence the lives of students at critical stages of development (Park and Hill, 2020). Students can gain interpersonal trust through perceived teacher reliability (Dong et al., 2020), interpersonal competence (Park and Hill, 2020) and initiative skills (Park and Hill, 2020), among other character traits, which influence their PB (Longobardi et al., 2021). It has been noted that there is a positive relationship between instructors’ character teaching behaviors and individual students’ character behaviors (Nejati and Shafaei, 2017). A study has found that teachers and students is also positively related with students’ PB (Longobardi et al., 2021). Therefore, improving teachers’ character teaching behaviors has is an effective way to improve students’ PB (Yao and Enright, 2022).

Social support is considered an important interpersonal factor influencing PB (You et al., 2020), which makes people feel cared for and respected, plays an important role in social solidarity and maintaining psychological well-being (Feeney and Collins, 2015), which is frequently used as a research factor in studies related to positive psychology (Greenglass and Fiksenbaum, 2009). Previous research has shown that social support from parents and peers or significant others is significantly and positively associated with PB. Such as, Mauer et al. (2022) surveyed 1,539 students at 11 universities in the United States and specified that there was a positive relation between social support and individual PB. Liu A. et al. (2021) specified that social support promotes PB and reduces levels of self-antisocial behavior. Again, Jessor and Turbin (2014) found that social support was associated with PB in a study of American and Chinese adolescents. Oh and Roh (2022) surveyed 480 employees in Korea and found a positive relation between PSS and PB. Based on this, the present study concluded that PSS has an important relationship with PB.

This study conducted students from higher vocational institutions in Guangxi, China in the framework of the higher vocational education in China’s development. Using the PB of higher education students to analyze students’ character education, four questions were proposed and address from of self-determination theory’s point of view: How does a SSC affect the PB of higher education students? Which variables mediate or moderate the relationship between feelings of social connectedness and PB? What are the internal mechanisms of influence? How can revealing the inner mechanisms help us progress the character of higher education students?

## Research hypothesis and theoretical framework

Deci and Ryan (1980) proposed Self-Determination Theory (SDT), which has been developed into Basic Psychological Needs Theory, Organic Integration Theory, Cognitive Appraisal Theory and Causal Orientation Theory (Peng et al., 2022). SDT suggests that behavioral motivation can be distributed into two key types: a “sense of being controlled” and “sense of autonomy.” Sense of autonomy refers to the possibility for individuals to decide what is best for them, while satisfaction with autonomy (i.e., a sense of social connectedness) contributes to students’ intrinsic motivation (Ryan et al., 2021), and a sense of control refers to imposed personal goals, including influences from outside (Benita and Matos, 2021), such as teacher character, social support, etc. Once the external environment satisfies the individual’s sense of belonging helps to motivate his or her intrinsic tendency to internalize and integrate, changing exterior rules and desires into internally shared values (Vansteenkiste et al., 2006). Perceived teacher character behavior is considered to be an important factor influencing students’ PB (Yao and Enright, 2022), and PSS has also been shown to be related to students’ PB (Oh and Roh, 2022). Self-determination theory is applicable to explain the influence of SSC on PB of college students. Therefore, this study investigates the association between SSC, perceived teacher character behavior, and PSS and PB based on self-determination theory.

### Sense of social connectedness and PB

Stronger social relationships (e.g., peer attachment, friendship quality) influence subsequent PB (Malonda et al., 2019; Porat et al., 2020; García Vázquez et al., 2021; Dryburgh et al., 2022). Therefore, students’ PB is influenced by social relationships (Preston and Rew, 2022). Bartlett and DeSteno (2006) found that people who have received help from others are more likely to provide monetary help to others. A sense of interpersonal social connectedness influences prosocial motivation and behavior (Ryan and Deci, 2000; Pavey et al., 2011; Peacock, 2020). Maiya et al. (2020) suggested that students’ low contact with peers, teachers, or other important people leads to a decrease in PB. It is known that PB of students is an influential factor in the development of social relationships (Batool and Lewis, 2020). Thus, it can be argued that students with a stronger SSC are more likely to involve in PB. Based on this view, the following hypothesis was proposed.

*H1*: Sense of social connectedness has a positive effect on PB.

### Mediating role of perceived teachers’ character teaching behaviors

The quality of the teacher-student relationship is an important factor influencing students’ sense of connectedness and it predicts higher levels of teacher character teaching behavior among students (Ramberg et al., 2019). Such as, Jaekel et al. (2021) found that students with a strong SSC perceive higher professionalism in teachers, while students with a weak SSC have lower expectations of the profession or are at increased risk for teacher moral hazard (Ozkilic and Kartal, 2012). Their study also found that students’ perceptions of their professionalism may be weaker than those of their peers. One study found a positive association between student–student connectedness and student-teacher connectedness (Stone and Logan, 2018; Bireda, 2019), so students are more probable to connect with their peers when teachers recognize their abilities and regularly engage in meaningful communication with them (Stone and Springer, 2019), and this positive teacher-student relationship in turn promotes perceived teacher character teaching behaviors (Jederlund and von Rosen, 2021).

Teachers are the most important factor in improving schools and promoting student growth (Morgado et al., 2017), and teachers’ character teaching behaviors are related to their teaching relationship strategies. Some researchers have argued that teachers’ views of character as demonstrated in practice are more influential than teachers specifically teaching character education lessons (Campbell, 2006; Willemse et al., 2008; Ehrich et al., 2011; Harvey et al., 2022). Students’ perception of the teacher’s character traits is closely related to the students’ character or PB (Tirri, 2002; Ollrogge et al., 2022). From a character education perspective, teachers should focus on respecting students’ different opinions (Wubbels et al., 2013; Frenzel et al., 2021). For example, by communicating equally in teacher-student interactions (Campbell, 2006). In addition, the teacher is the leader of the class and the students act as followers as the teacher models and discusses character in order to stimulate changes in certain values, beliefs and other cognitive structures of character in the students (Gardner et al., 2005; Mulyaningsih et al., 2022). Similarly, students reduce differences in values and behaviors with their teachers (Avolio and Gardner, 2005), resulting in PBs (Blasi, 1980). Therefore, this study suggests that the teacher’s role in the classroom is similar to that of a leader and that students influence their own PBS by perceiving the teacher’s moral behavior.

Aknin et al. (2013) suggested that promoting a SSC, friends or acquaintances receive the greatest emotional rewards and generate positive social interactions (Williamson and Clark, 1989). For example, a stronger SSC among students was positively associated with teacher moral performance (Ramberg et al., 2019; Jaekel et al., 2021). In addition, teachers have a modeling role for students in their classes, and different teachers have different patterns of teaching behaviors and dealing with students’ behavioral expressions (Asif et al., 2020). Good teacher character teaching behaviors also influence students to develop good character (Cheon et al., 2018; Popovska and Popovski, 2021), and when teachers’ teaching behaviors have character characteristics such as reliability, initiative and good interpersonal relationships (Park and Hill, 2020), which can influence students to exhibit more PB (Hannah et al., 2015; Fogelgarn et al., 2021). Thus, teachers who want to improve students’ PB should first improve their own moral character. Therefore, it is reasonable to speculate that teachers’ moral teaching behaviors mediate the association between students’ SSC and their PBs. From the discussion, the hypotheses are proposed as follows:

*H2*: A sense of social connectedness has a positive effect on perceived teacher character teaching behavior.

*H3*: Perceived positive effects of teachers’ character teaching behaviors on PBs.

*H4*: Teachers’ character teaching behavior mediates the relationship between students’ sense of social connectedness and their PB.

### Mediating role of PSS

A SSC is considered to be an inherent sense of intimacy with the social world, including relationships with family, friends, neighborhoods, schools and communities (Hashemi et al., 2020), and access to available resources for individuals through social connectedness (Ozbay et al., 2008). Similarly, PSS can have characteristics such as virtual, and is often studied in terms of technical support, emotional support, spiritual support and social connectedness with others (Waite, 2018). According to the three basic psychological needs of SDT “autonomy, competence and relatedness” noted that when the social environment supports these basic needs, it can promote psychological growth and produce positive behaviors (Ryan and Deci, 2017). The connectedness of exchanging support between individuals can satisfy the need for emotional connectedness in society (Hashemi et al., 2020; Xu et al., 2021). Research has demonstrated a positive relationship between perceived social connectedness and perceived support from family, teachers and significant others (Hornstra et al., 2021; Tüzün et al., 2022). For example, not only does perceived social connectedness promote PSS, but PSS also promotes perceived social connectedness.

PSS plays a critical role in sustaining psychological well-being (Hornstra et al., 2021). Moreover, Taylor (2011) argued that PSS is a feeling of being a valued member of a social network where people feel cared for and expect help when they need it. For example, family, friends and others provide emotional and financial help (Arifuddin et al., 2022), and this help helps to enhance the effectiveness of coping strategies (Yildirim and Arslan, 2020). In other words, social support has many positive functions (Costa-Cordella et al., 2021). Many research has indicated that social support from significant others (e.g., parents and peers) is also significantly associated with PB (Esparza-Reig et al., 2021; Haller et al., 2021; Hu et al., 2021). For example, Prince et al. (2019) found that social resources were a predictor of PB in adolescents. Rosenfeld et al. (2000) found that students who received more social support exhibited more PB than students who did not receive more social support.

In previous studies, SSC and social support were not always considered separately (Asante and Karikari, 2022). For example, the positive effects of SSC and social support on adolescents’ mental health and well-being (Arslan, 2018; Çiçek, 2021). A SSC has been shown to enhance social support and reduce loneliness (Duru, 2008; Reyes et al., 2020). That is, better interpersonal relationships with others result in more social support (Ozbay et al., 2008; Alegre, 2012), which promotes PB (Barry and Wentzel, 2006). In addition, PSS includes support from significant others, family and friends (Zimet et al., 1990), and the higher the degree of perceived support from significant others such as family, friends, and teachers, the more PBs are generated at school and home. Which are the primary sites of student activity (Shin, 2021). In a study of 492 students in China, (Liu C. et al. 2021) found that increased social support facilitated PB and decreased antisocial behavior (Lenzi et al., 2012). Such as PSS was positively related to PB (Haller et al., 2021; Smith and Crosby 3rd., 2021).

A recent study showed that teachers’ enthusiasm is contagious in the classroom and positively affects students’ emotions (Dewaele and Li, 2021), and that enthusiastic teaching not only motivates and inspires learners, but also enhances students’ PSS (Camacho et al., 2021; Zhao and Qin, 2021). How do teachers’ character teaching behaviors affect students’ PSS? Explained according to cognitive assessment theory under SDT (Deci and Ryan, 1985), social context and key social factors are critical to meeting students’ basic psychological needs for autonomy, competence and relatedness. In the school setting, teachers are the key (Peng et al., 2022) social factor and social context of the school, and teachers have a greater impact on students in school than any other factor in the school (Viano et al., 2020). For example, students feel safer at school when teachers’ positive teaching behaviors affect student autonomy (Coyle et al., 2021) and thus feel socially supported (Ahn et al., 2021). According to the above discussion, this study concluded that teachers’ character teaching behaviors can positively influence students’ PSS. Therefore, it is practical to say that PSS mediates between the SSC and PB and the following hypotheses is proposed:

*H5*: A sense of social connectedness has a positive effect on PSS.

*H6*: PSS has a positive effect on PB.

*H7*: PSS mediates the relationship between a sense of social connectedness and PB.

*H8*: Teachers’ character teaching behaviors have a positive effect on PSS.

*H9*: Sense of social connectedness can indirectly predict students’ PB through the linked mediating role of teachers’ character teaching behaviors and PSS.

**Figure 1:**
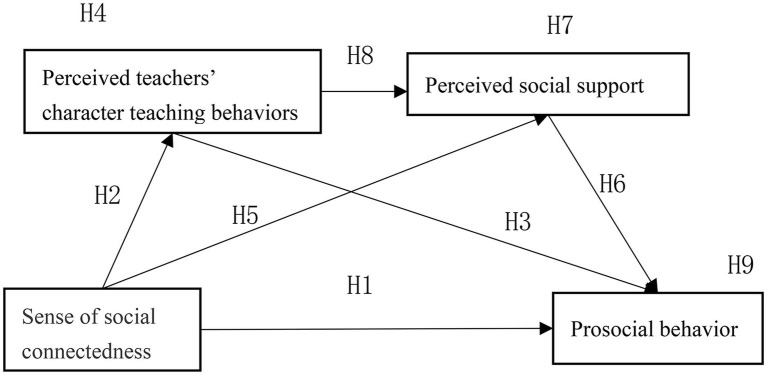
A theoretical model showing the influence of sense of social connectedness on the PB of higher education students*.*

On the basis of H1–H9, hypothetical model was proposed showing the influence of the sense of social connectedness on the PB of higher education students (Figure 1).

## Methodology

### Participants and procedures

This study was investigated in April 2022. We selected 10 full-time colleges and universities in Guangxi, China for the study, and distributed to each school using a convenience sampling method with wjx.cn, a Chinese questionnaire platform. A total of 1,150 questionnaires were retrieved, and after eliminating 87 invalid questionnaires with short response times, 1,007 valid questionnaires were used for analysis, with a valid response rate of 92.43%. The demographic information of the participants is as follows: in terms of gender distribution, most of the sample is male, accounting for 67.1%, and the female sample is 32.9%. In terms of grades, the sample has relatively more 2021 classes, with a proportion of 48.2, and 35.7% are 2020 classes, and 14.3% are 2019 classes.

### Measurements

The four scales, Sense of Social Connectedness Scale, the PSS Scale, Perceived Teachers’ Character Teaching Behaviors Scale and the PB Scale, were initially developed in English and then a translation into Chinese were done for the purpose of this study. To improve the precision of the translation, a reverse translation method was implemented (Brislin, 1970). The first translator made an initial translation from English scales into Chinese. The second translator translates the translated scale back into English, and then the third translator combines the original scale, the translated scale, and the back-translated scale. All three versions of the questionnaire were then compared, and the translations revision were made to confirm the equivalence of the scale content. At the same time, 15 Chinese higher education students were invited to test-complete the questionnaire in this study, aiming to focus on reviewing the textual description, the completeness of the connotation and the fluency and comprehension of the text, of each question aspect of the questionnaire, and finally based on the feedback the revision of the textual was made. The questionnaire content was assessed using a “5-point Likert scale: 1 = indicates strongly disagree, 2 = indicates disagree, 3 = indicates fair, 4 = indicates agree, and 5 = indicates strongly agree.”

This study used questionnaires to investigate college students’ SSC, teachers’ character teaching behaviors, PSS and PBs in higher education. The reliability and validity of the scales were analyzed using AMOS (v.23.0, IBM,), SPSS (v.25.0, IBM). Cronbach’s alpha>0.7 (Gefen et al., 2000). To ensure the model fit, first-order validation factor analysis was used to analyze the items for each scale to ensure a good fit, and the *χ*^2^/df value should smaller than 5; root mean square error of approximation (RMSEA) should be smaller than 0.10; Goodness-of-Fit Indices (GFI) and Absolute Goodness-of-Fit Indices (AGFI) must be greater than 0.80; the convergent validity of the item is established by the factor loading values and it is suggested to remove when the value is below 0.50 (Kenny et al., 2015). The AVE should be greater than 0.5, and if the AVE is smaller than 0.5 but the composite reliability is greater than 0.6, it indicates that the convergent validity construct is still met (Fornell and Larcker, 1981). Model 6 in the SPSS macro program developed by Hayes (2012) ^1^ was used to conduct the mediating role of perceived teacher character teaching behaviors and PSS in the association between SSC and PB.

The SSC was analyzed using the Social Connectedness Scale (SCS-R) developed by Lambert et al. (2013). The scale is unidimensional and consists of seven items, all of which are scored on a five-point Likert scale, with 1 being “completely disagree” and 5 being “completely agree,” and as the score increases, the college student’s SSC increases, and the participant must choose one of five options from completely agree to completely disagree, such as “I am happy to share what I have with my friends.” The Cronbach’s alpha for the scales was 0.708, representing that the scales have good reliability (CFI = 0.935, TLI = 0.903, RMSEA = 0.094, RMR = 0.033, SRMR = 0.041, GFI = 0.962, AGFI = 0.924, CR value = 0.818 and AVE = 0.393). The CR and AVE met the threshold value and the factor loadings of each item ranged from 0.556 to 0.727. The scale has great convergent validity and structural validity.

Perceived teacher character teaching behaviors were measured using Park and Hill’s (Park and Hill, 2017) Occupational work ethic inventory, which is structured with three main dimensions of interpersonal skills, initiative, and reliability, consisting of 12 words or two-word descriptors: friendly, cheerful, likeable, courteous, perceptive, effective, ambitious, resourceful, following instructions, compliant and reliable. All items on the scale were measured on a five-point Likert scale. Where 1 means not “completely disagree” and 5 means “completely agree.” As scores increased, the level of perceived teacher character teaching behaviors increased. The Cronbach’s alpha of the scales was 0.911, 0.899 and 0.879, respectively, indicating good reliability of the scales (CFI = 0.948, TLI = 0.933, RMSEA = 0.104, SRMR = 0.034, RMR = 0.025, GFI = 0.895 and AGFI = 0.840, CR values = 0.903, 0.900 and 0.856, AVE = 0.700, 0.648 and 0.664). The CR and AVE met the threshold value and the factor loadings of each item ranged from 0.714 to 0.878. The scale has good convergent validity and structural validity.

PSS was analyzed with Multidimensional Scale of Perceived Social Support developed by Zimet et al. (1990). The scale was used to analyze social support among American adolescents, the scale has evolved into one of the most widely used social support outcome measures, which focuses on PSS by measuring the amount of social support an individual receives from three sources: friends, family, and significant others. The scale is divided into 3 dimensions and 12 items. Important Other People Support includes four items, such as “There are people (leaders, relatives, classmates) who are there for me when I have problems.” Friend support consists of four items, including “My friends can really help me.” Family support includes four items such as “My family can help me in a concrete way.” Similarly, items were assessed using a five-point Likert scale, where 1 means not “completely disagree” and 5 means “completely agree.” As scores increased, the level of PSS also increased. The Cronbach’s alpha for the three dimensions were 0.917, 0.891 and 0.921, respectively, showing good reliability. Latent variable models were developed for all three sub-dimensions of the scale, and the model fit indices were again good [CFI = 0.921, TLI = 0.939, RMSEA = 0.090, SRMR = 0.042, RMR = 0.030, GFI = 0.921 and AGFI = 0.880, CR values = 0.892, 0.871 and 0.901, AVE values = (0.674, 0.629 and 0.695)]. The CR and AVE met the threshold value and the factor loadings for each item ranged from 0.580 to 0.874. Therefore, the study scale has great convergent and structural validity.

PB was measured using Adult PB Scale developed by Caprara et al. (2005). The main feature of PB is the ability to distinguish individual differences in PB among adults and is a very widely used recent measure of PB (Martinez-Pampliega et al., 2018). The Adult Prosociality Instrument contains 16 prosocial items on four dimensions: “sharing with others, helping, caring, and empathizing with others’ feelings.” Sharing with others includes seven items, such as “I am happy to share what I have with my friends.” Helping others includes three items, such as “I can help those who need help in a timely manner.” Caring for others includes three items, such as “I try to comfort those who are sad.” Empathizing with the feelings of others included three items, such as “I empathize with those who really need help.” The items were measured using a five-point Likert scale, the same as the previously described scale. As the total score obtained increased, the more PB was produced. The results of the test showed good reliability with Cronbach’s alpha of 0.906, 0.877, 0.888 and 0.800 for the four dimensions, respectively (CFI = 0.896, TLI = 0.873, RMSEA = 0.114, SRMR = 0.052, RMR = 0.033, GFI = 0.811 and AGFI = 0.738, CR values = 0.887, 0.845, 0.861 and 0.746, AVE values = 0.533, 0.646, 0.674 and 0.495). The CR and AVE met the threshold value, and the factor loadings of each item ranged from 0.511 to 0.858. Therefore, the study scale has great convergent and structural validity.

## Results

### Descriptive statistics and correlation analysis

Table 1 indicates the general means, standard deviations and correlation coefficients for SSC, perceived teacher character teaching behavior, PSS and PB. The results indicate that all variables are significantly correlated. SSC was positively related with perceived teacher character teaching behavior and PB (*r* = 0.325, *p* < 0.001; *r* = 0.542, *p* < 0.001; *r* = 0.471, *p* < 0.001). Perceived teacher character teaching behavior was positively related with PSS and PB (*r* = 0.409, *p* < 0.001; *r* = 0.425, *p* < 0.001). PSS was significantly and positively associated with PB (*r* = 0.666, *p* < 0.001).

**Table 1:** Means, standard deviations, and correlations of the main study variables Pearson correlation.

Scale	M	SD	1	2	3	4
SSC	3.576	0.586	1			
Perceived teachers’ character teaching behaviors	4.289	0.676	0.325**	1		
PSS	3.805	0.654	0.542**	0.409**	1	
PB	3.961	0.584	0.471**	0.425**	0.666**	1

** *p* < 0.01.

SSC, Sense of social connectedness; PSS, Perceived social support; PB, Prosocial behavior.

### Chain mediation model analysis

SSC, perceived teacher character teaching behavior, PSS and PB were significantly correlated and met the statistical requirement for analyzing the mediating effects of perceived teacher character teaching behavior and PSS. Model 6 in the SPSS macro program developed by Hayes (2012) ^2^ was used to conduct the mediating role of perceived teacher character teaching behaviors and PSS in the association between SSC and PB.

Table 2 indicates the results of the regression analysis of the relationship between the SSC and PB. The outcomes showed that the SSC has a positive significant predictive effect on PB (*B* = 0.471, *p* < 0.001). SSC was a significant positive predictor of perceived teacher character teaching behavior (*B =* 0.325, *p* < 0.001), SSC was a significant positive predictor of PSS (*B* = 0.542, *p* < 0.001), perceived teacher character teaching behavior was a significant positive predictor of PSS (*B* = 0.409, *p* < 0.001), and perceived teacher character teaching behavior was a significant positive predictor of PB (*B* = 0.425, *p* < 0.001), PSS was a significant positive predictor of PB (*B* = 0.666, *p* < 0.001), and hypotheses H1–H5, H8 were tested. Perceived social connectedness significantly predicted perceived teacher character teaching behavior (*B* = 0.325, *p* < 0.001) and PSS (*B* = 0.458, *p* < 0.001) when perceived teacher character teaching behavior and PSS were included in the regression equation. Perceived teacher character teaching behaviors significantly predicted PSS (*B* = 0.260, *p* < 0.001) and PBs (*B* = 0.167, *p* < 0.001). In addition, PSS was a significant positive predictor of PB (*B* = 0.526, *p* < 0.001). Currently, the value of the direct effect of the SSC on PB was significantly lower (*B* = 0.132, *p* < 0.05). These results suggest that the chain of perceived teacher character teaching behaviors, PSS, and perceived teacher character teaching behaviors → PSS mediated significantly in the effect of SSC on PB. Hypotheses H6, H7, H9 are confirmed.

**Table 2:** Regression analysis of the relationship between sense of social connectedness and prosocial behavior.

Result variables	Predictive variables	Fit index			Significance	
		*R*	*R* ^2^	*F*	*B*	*t*
PB	SSC	0.471	0.222	286.552	0.471	16.928^***^
Perceived teachers’ character teaching behaviors	SSC	0.325	0.106	118.536	0.325	10.887^***^
PSS	SSC	0.542	0.294	418.056	0.542	20.446^***^
PSS	Perceived teachers’ character teaching behaviors	0.409	0.167	201.348	0.409	14.190^***^
PB	Perceived teachers’ character teaching behaviors	0.425	0.180	220.937	0.425	14.864^***^
PB	PSS	0.666	0.443	798.974	0.666	28.266^***^
PSS	SSC	0.595	0.354	275.332	0.458	17.064^***^
	Perceived teachers’ character teaching behaviors				0.260	9.692^***^
PB	SSC	0.695	0.483	312.225	0.132	4.833^***^
	Perceived teachers’ character teaching behaviors				0.167	6.646^***^
	PSS				0.526	18.613^***^

*** *p* < 0.001.

SSC, Sense of social connectedness; PSS, Perceived social support; PB, Prosocial behavior.

**Figure 2:**
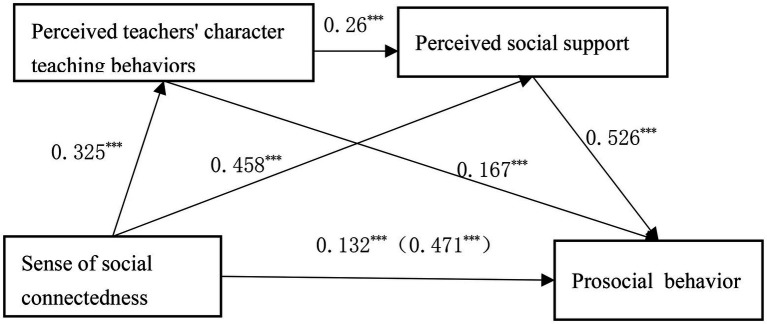
Chain mediation model. ***P<0.001.

Table 3 indicates the mediated effect values of perceived teacher character teaching behavior and PSS between SSC and PB. Figure 2 shows the chain mediation model between perceived social connectedness and PB. Table 3; Figure 2 show that perceived teacher character teaching behaviors and PSS mediated significantly between feelings of social connectedness and PBs, with a total standardized mediation effect value of 0.773. Specially, the mediating effect consisted of three indirect effects: path one—SSC → perceived teacher character teaching behavior → PB (0.124), path two—SSC → PSS → PB (0.548), and path 3—SSC → perceived teacher moral teaching behavior → PSS → PB (0.101). The ratio of the three indirect effects to the total effect is 11.55, 51.08, and 9.41% for the three paths 1, 2, and 3 the 95% confidence interval (CI) for these indirect effects does not contain a zero value, signifying that all three indirect effects met a significant level. Thus, hypotheses H6, H7 and H9 are again confirmed. The bootstrapped 95% CI for the difference of the indirect effects 1 and 2 in comparison 1 does not contain a value of 0, showing a significant difference in them. Using the same method, a significant difference was found in indirect effects 2 and 3 in comparison 3, while there were no significant differences in the bootstrapped 95% CI package 0 values between indirect effects 2 and 3 in comparison 2. These findings suggest that a SSC not only indirectly predicts PB through a single mediating effect of perceived teacher character teaching behavior or PSS, but also through a chain mediating effect of perceived teacher character teaching behavior and PSS. The single mediating effect of PSS accounted for the highest proportion of the total effect (51.08%).

**Table 3:** The role of Perceived Teachers’ Character Teaching Behaviors and perceived social suppor in the analysis of mediating effects.

	Indirect effects	Boot SE	Boot LLCI	Boot ULCI	Relative mediating effect
Total indirect effect	0.773	0.053	0.674	0.879	72.07%
Indirect effects1	0.124	0.026	0.076	0.178	11.55%
Indirect effects12	0.548	0.05	0.453	0.652	51.08%
Indirect effects13	0.101	0.017	0.07	0.139	9.41%
Comparison 1	−0.425	0.064	−0.549	−0.3	
Comparison 2	0.022	0.027	−0.029	0.077	
Comparison 3	0.447	0.053	0.345	0.554	

Chain mediated model showing the effects of SSC, perceived teacher character teaching behaviors, and PSS on prosociality *n* = 1,007. Values in parentheses are the direct effect of the SSC. The regression coefficients were obtained in the PROCESS program of SPSS.

*** *p* < 0.001.

## Discussion

The findings of this study suggest that the mediating role of perceived teacher character teaching behaviors and PSS may contribute to understanding the relationship between feelings of social connectedness and PBs in a sample of Guangxi, China, higher education students.

### The relationship between the sense of social connectedness and PB

First, consistent with previous research (Zhu et al., 2020), this study found that SSC significantly and positively predicted PB among senior students in Guangxi, China. That is, the higher the level of social connectedness, the more likely it is that PB will occur and produce more PB. This result implies that the SSC in the Chinese context is an important factor in the occurrence of PB among higher education students. Close social ties enhance people’s perception of their own value and sense of belonging. Thus increasing their sense of competence and autonomy to generate PB, which is important for promoting positive contributions to society in the long run.

### Sense of social connectedness, perceived relationship between teachers’ character teaching behaviors and PBs

While previous research has confirmed that a SSC can influence PB, how does a SSC influence PB? The internal process mechanisms are not clear. Previous research has found that a SSC can lead to self-esteem and thus promote PB in students (Frick et al., 2021; Preston and Rew, 2022). This study further investigated the relation between perceptions of social connectedness and PB from a different perspective. For the first time, we found that perceived teacher character teaching behaviors were a mediating variable of social connectedness affecting PB. This finding implies that a SSC can facilitate PB by enhancing perceived teacher character teaching behaviors. China is known as the land of etiquette and has more complex cultural characteristics of interpersonal social interaction, and higher education students are at an important developmental milestone (Van et al., 2021), which is extremely important to promote students’ social communication and enhance interpersonal etiquette (Li et al., 2021). In such a context, it is extremely important to enhance the sense of social connection. By enhancing students sense of connectedness, higher education students will be able to integrate into their surroundings and adapt to life more quickly, and will be able to act in a socially beneficial manner. The finding that teachers are important actors in schools and role models for students’ character learning, and that perceiving teachers’ character teaching behaviors, students also become more motivated to produce PBs, further explains the moral identity theory perspective (Weaver, 2016).

### The relationship between sense of social connectedness, PSS, and PB

This study found that PSS is a significant mediating variable in the mechanism by which social connectedness affects PB of our higher education students. Previous research has demonstrated that PSS mediates the influence of social connectedness on PB, i.e., people with a higher SSC are more likely to perceive social support, and these people are more likely to engage in PB, such as participating in epidemic volunteer activities (Okabe-Miyamoto and Lyubomirsky, 2021). This suggests that a SSC can promote PB by enhancing PSS. First, we found that a SSC can facilitate PSS. Previous research has shown that a SSC can alter negative interpersonal perceptions and can increase students’ PSS (Dozois, 2021), and our study confirms this relationship. Unlike secondary schools or undergraduate institutions, higher education schools mainly focus on developing students’ vocational skills and neglect the development of students’ sense of belonging and social support (Pu, 2020). Therefore, higher vocational students need to pay more attention to social interpersonal interactions in their daily study and integrate into the needs of the social environment, so that they can perceive their own value and be willing to have more patience and responsibility. At the same time, a SSC ensures one’s integration into the social interaction environment and experiences a sense of belonging. A high level of belonging increases PSS, further increasing the incidence of PB in students. An increase in PSS means an increase in psychological identity for higher education students, generating a strong sense of belonging and loyalty is important for stimulating PB generation.

### The relationship between sense of social connectedness, perceived teacher character teaching behavior, PSS, and PB

For the first time, we found that the chain mediated by perceived teacher character teaching behavior → PSS is also an important way in which the SSC influences PB among our higher education students. This finding implies that a SSC can enhance higher education students’ perceived teacher character teaching behaviors, and perceived teacher character teaching behaviors can promote PSS, which further promotes PBs. This finding also indicates the relative complexity of the process by which the SSC affects PB. Although our study found independent mediating effects for perceived teacher character teaching behaviors and PSS, as well as a chain of mediating effects formed by them, they both produced partial mediating effects. Therefore, it is not possible to completely clarify the association between the SSC and PB. In fact, there may be other factors at play, which deserve further study. In addition, we found that among all the paths through which SSC influences PB of higher education students, the path of SSC → PSS → PB has the utmost indirect effect value, accounting for 51.08% of the total effect. This result indicates that PSS is very important for our higher education students and is an important factor in inducing PB. Thus, this study argues that we must pay more consideration to the development of PB cultivation of higher vocational students.

## Findings, significance and limitations of The study

### Conclusion

This study found that the SSC not only directly predicted students’ PB, but also indirectly predicted students’ PB through the independent mediating roles of perceived teacher character teaching behavior and PSS. It is also possible to indirectly predict students’ PB through the interlocking mediating effects of perceived teacher character teaching behavior and PSS.

### Significance of the study

This study’s theoretical contributions include the following: first, this study reveals the direct effects of the SSC on perceived teachers’ character teaching behaviors, PSS, and PBs. Ryan and Deci (2000) found that self-determination theory can be divided into two dimensions: intrinsic motivation, external factors, and SSC as an internal factor. Which has also been found that when a person feels included in a group, experiences a sense of connectedness to a group or community, and has close relationships with others, this need is met and PB results. When a close relationship is established, this need is satisfied and PB is generated. The influence of teachers’ character teaching behaviors and social support as external factors on PB. The results of the study enriched the self-determination theory in PB. Second, this study examines the development and emerge of PB from the SSC point of view, whereas previous studies have mainly analyzed it from the perspective of peers, attachment, and psychological capital, broadening the analytical perspective of PB research. Third, this study creatively incorporated factors such as perceived teacher character teaching behavior and PSS into a theoretical model of the effect of a SSC on PB. Perceived teacher character teaching behaviors and PSS were found to have a chain mediating effect on PBs in higher education students’ SSC, which has not been reported in previous studies. Therefore, this study offers a theoretical reference for China to solve the problems faced by higher education such as the lack of PB of students.

This study can offer understandings for effectively improving PBs of higher education students in practice. First, the SSC can directly predict the PB of higher education students. Therefore, the attention of cultivation of students’ sense of social connection should be paid to. Both can enhance their sense of belonging to social interpersonal interactions through social connection and to enhance the occurrence of PB through a sense of close connection to society. Second, the SSC can influence the production of PBs of higher education students through perceived teachers’ moral teaching behaviors, PSS and the chain mediation between these two factors, indicating that perceived teachers’ moral teaching behaviors and PSS are the key factors influencing higher education students’ PBs. On one hand, the cultivation of teachers’ character teaching behaviors and enhancing their character traits can be divided into three aspects: interpersonal interaction, initiative and reliability, so as to enhance the influence of teachers’ character teaching behaviors on students. On the other hand, more attention should be focused on higher vocational students’ perceptions and attitudes toward the sense of social connection. Only by helping them to have positive perceptions and beliefs about interpersonal interactions can the incidence of PB be increased.

### Limitations of the study

There was also some limitation in this study. First, due to spatial and temporal constraints, this study used a cross-sectional research design. While preceding research have delivered the basis for this type of study, there are still some difficulty to derive same causal relationships by means of this method. Future studies, may consider experimental or longitudinal methods to further examine the causal relationships of different variables. Second, the findings of this study cannot be applied to all higher education students. The population of this study was senior vocational students in Guangxi, China, and 48.2% were first-year senior vocational students, while only 14.3% were third-year students; therefore, the grade distribution ratio was unbalanced. Future studies, should consider the proportion of participants’ grades and the PBs produced by higher vocational students in different countries or regions may be studied for comparison. Third, because this study used a self-reported questionnaire, it is challenging to rule out the probability that participants’ responses were exaggerated or conservative when in comparison to the real-life condition. Thus, future studies can collect data from various sources of information.

## Data availability statement

The original contributions presented in the study are included in the article/supplementary material, further inquiries can be directed to the corresponding author.

## Ethics statement

Ethical review and approval was not required for the study on Human Participants in accordance with the Local Legislation and Institutional Requirements. Written informed consent from the participants was not required to participate in this study in accordance with the National Legislation and the Institutional Requirements.

## Author contributions

All authors listed have made a substantial, direct, and intellectual contribution to the work and approved it for publication.

## Funding

This study was supported by “Research topics on the theory and practice of ideological and political education for college students in Guangxi”. (grant no. 2021LSZ078).

## Conflict of interest

The authors declare that the research was conducted in the absence of any commercial or financial relationships that could be construed as a potential conflict of interest.

## Publisher’s note

All claims expressed in this article are solely those of the authors and do not necessarily represent those of their affiliated organizations, or those of the publisher, the editors and the reviewers. Any product that may be evaluated in this article, or claim that may be made by its manufacturer, is not guaranteed or endorsed by the publisher.

## Footnotes

^1^ http://www.afhayes.com

^2^ http://www.afhayes.com
